# Liver Fibrosis and MAFLD: From Molecular Aspects to Novel Pharmacological Strategies

**DOI:** 10.3389/fmed.2021.761538

**Published:** 2021-10-22

**Authors:** Weiyi Qu, Tengfei Ma, Jingjing Cai, Xiaojing Zhang, Peng Zhang, Zhigang She, Feng Wan, Hongliang Li

**Affiliations:** ^1^Department of Cardiology, Renmin Hospital of Wuhan University, Wuhan, China; ^2^Institute of Model Animal, Wuhan University, Wuhan, China; ^3^Department of Neurology, Huanggang Central Hospital, Huanggang, China; ^4^Huanggang Institute of Translational Medicine, Huanggang Central Hospital, Huanggang, China; ^5^Department of Cardiology, The Third Xiangya Hospital, Central South University, Changsha, China; ^6^School of Basic Medical Sciences, Wuhan University, Wuhan, China

**Keywords:** liver fibrosis, metabolic associated fatty liver disease, drug target, non-alcoholic fatty liver disease, non-alcoholic steatohepatitis, cirrhosis

## Abstract

Metabolic-associated fatty liver disease (MAFLD) is a new disease definition, and this nomenclature MAFLD was proposed to renovate its former name, non-alcoholic fatty liver disease (NAFLD). MAFLD/NAFLD have shared and predominate causes from nutrition overload to persistent liver damage and eventually lead to the development of liver fibrosis and cirrhosis. Unfortunately, there is an absence of effective treatments to reverse MAFLD/NAFLD-associated fibrosis. Due to the significant burden of MAFLD/NAFLD and its complications, there are active investigations on the development of novel targets and pharmacotherapeutics for treating this disease. In this review, we cover recent discoveries in new targets and molecules for antifibrotic treatment, which target pathways intertwined with the fibrogenesis process, including lipid metabolism, inflammation, cell apoptosis, oxidative stress, and extracellular matrix formation. Although marked advances have been made in the development of antifibrotic therapeutics, none of the treatments have achieved the endpoints evaluated by liver biopsy or without significant side effects in a large-scale trial. In addition to the discovery of new druggable targets and pharmacotherapeutics, personalized medication, and combinatorial therapies targeting multiple profibrotic pathways could be promising in achieving successful antifibrotic interventions in patients with MAFLD/NAFLD.

## Introduction

Due to the close association with metabolic disorders and the previous exclusionary diagnostic strategy facing many challenges, a new disease nomenclature, metabolic-associated fatty liver disease (MAFLD), was proposed by expert panels to renovate its former name, non-alcoholic fatty liver disease (NAFLD) (1, 2). However, there are committees and experts who believe that the molecular basis of the disease behind this new definition lacks sufficient understanding, which may lead to uncertainty and negative effects in this field (3). Although many aspects of MAFLD are not well-understood, the similarities of the prevalence, risk factors, and pathological and metabolic traits between MAFLD and NAFLD suggest that evidence from NAFLD over the past decades would provide valuable clues for the discovery of druggable targets for the treatment of MAFLD and its subsequent fibrosis (4–7).

Non-alcoholic fatty liver disease is the most common chronic liver disease globally and affects approximately a quarter of the world population (5, 8, 9). It progresses from simple liver steatosis to nonalcoholic steatohepatitis (NASH) and, in more severe cases, to liver fibrosis and cirrhosis (10). By 2030, the overall number of cases of this disease is projected to increase by 18.3%, and the number of cases of its related advanced liver disease and liver-related mortality will be doubled (11). Facing such a severe public health burden, MAFLD as a new concept still lacks direct and strong evidence from pharmaceutical investigations. Even for NAFLD with sufficient research data to endorse, there are no specific drugs approved by the United States Food and Drug Administration (US-FDA) or the European Medicines Agency (12, 13).

Fibrosis is a consequence of advanced liver injury that is closely associated with cirrhosis and liver carcinoma (14). Therefore, the improvement of liver fibrosis has become an important indicator for evaluating the efficacy of drugs for the treatment of NAFLD. However, the effectiveness of current drugs for hepatic fibrosis is limited (15). The identification of druggable targets and the development of novel reagents for the prevention and reversal of fibrosis will be an important mission of NAFLD/MAFLD research (16). This review summarizes the key endogenous molecules involved in the pathogenesis of NAFLD/MAFLD fibrosis and discusses the compounds or antibodies derived from these druggable targets that could potentially lead to successful treatments for NAFLD/MAFLD (Supplementary Table 1).

## Pathogenetic Mechanisms Underlying Fibrosis in MAFLD

**Figure 1:**
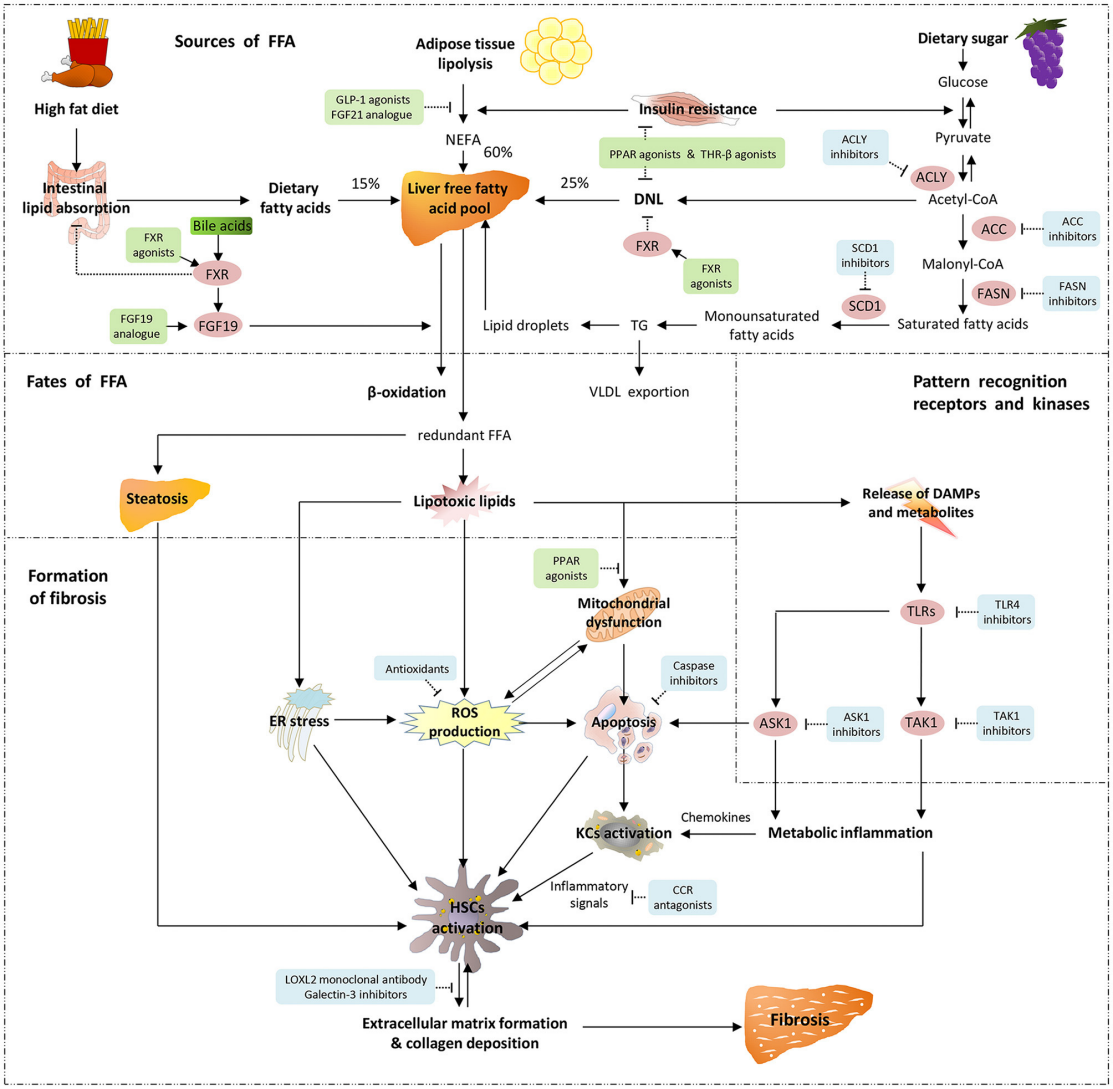
Pathogenetic mechanisms underlying fibrosis in MAFLD/NAFLD and molecular target of drug therapy. There are three sources of hepatic free fatty acids (FFA): 60% from the adipose tissue lipolysis or non-esterified fatty acid (NEFA) pool, 25% from *de novo* lipogenesis (DNL), and the remaining 15% from the intestinal absorption of diet. The two main metabolic pathways of hepatic FFA are mitochondria-mediated β-oxidation and esterification to form triglyceride (TG). Triglyceride is able to be exported into the circulation in the form of very-low-density lipoprotein (VLDL), and the excessive TG are stored in lipid droplets. When FFA are overaccumulated or their disposal is not timely, the redundant FFA act as substrates to produce lipotoxic lipids, which lead to endoplasmic reticulum (ER) stress, production of reactive oxygen species (ROS), impaired mitochondrial function and release of danger-associated molecular patterns (DAMPs). Pattern recognition receptors such as toll-like receptors (TLRs) sense the continuous production of DAMPs and metabolites, thereby triggering downstream signaling pathways. Apoptosis signal-regulating kinase 1 (ASK1) and TGF-β-activated kinase 1 (TAK1) are crucial intracellular signal transduction components that are activated by post-transcriptional modification, and further activate their downstream pivotal kinases and transcription factors, leading to the occurrence of metabolic inflammation. As metabolic stress leads to the expression and release of inflammatory chemokines, Kupffer cells (KCs) polarize into pro-inflammatory phenotypes and participate in the activation of hepatic stellate cells (HSCs) in coordination with ROS, apoptotic signals and ER stress. These exacerbate extracellular matrix formation and collagen deposition, and result in liver fibrosis. Emerging therapeutic agents and their molecular targets for fibrosis in MAFLD/NAFLD are also indicated. Agonists and analogs are marked in green, while antagonists, inhibitors and antibodies are marked in blue.

Fibrosis is the primary histological feature of the advanced form of NAFLD/MAFLD (17). Therefore, it is critical to elucidate the mechanism mediating liver fibrosis in NAFLD/MAFLD. Although the majority of the mechanistic discoveries were based on NAFLD, the new terminology MAFLD shares similar driving factors as NAFLD and knowledge from NAFLD provides important implication in the understanding of the pathogenesis of MAFLD. The pathophysiology of NAFLD progression is summarized as the “multiple hits” theory. That is, the “first hit” begins with hepatic triglyceride accumulation, and the responses of insulin resistance (IR)-related lipotoxic substances, and the increased *de novo* hepatic lipogenesis, thereafter oxidative stress, metabolic inflammation, endoplasmic reticulum stress, and autophagy together with the intestinal microbial signals and other links all involved, finally facilitating “parallel, multiple hits” to the liver (18). In the liver injury-repair process, dysregulated hepatocytes or inflammatory cells elicit paracrine signaling that promotes the hepatic stellate cells (HSCs) activation. Meanwhile, circulating factors (e.g., adipokine and fatty acids) from extrahepatic tissues (e.g., visceral adipose tissue or intestine) could activate HSCs directly or mediately (17). In addition, gene polymorphisms, such as PNPLA3, TM6SF2, and HSD17B13, may increase an individual's susceptibility to liver fibrosis during metabolic dysregulation (17, 19, 20). Upon the stimulation of the abovementioned profibrotic factors, HSCs turn into an active form and accelerate the production of fibroblasts, portal vein fibroblasts, and myofibroblasts, which ultimately result in exacerbated extracellular matrix formation (21). Overall, HSCs activation is a dominant manifestation during fibrosis in NAFLD (17, 21) (Figure 1).

As the main target of liver injury, hepatocytes first face the imbalance of fatty acid and carbohydrate metabolism caused by metabolic overload in the early stage of NAFLD (22). With the further development of hepatic steatosis, excess, or not timely disposed fatty acids could be metabolized into toxic lipids (such as oxidized phospholipids), causing hepatocyte metabolic stress and damage or death (22). This lipotoxic response leads to hepatocytes apoptosis, which liberates reactive oxygen species (ROS) and free cholesterol (17). Damaged hepatocytes serve as a major driver for HSCs activation via paracrine signaling. For example, lipotoxic associated ROS production from mitochondria, endoplasmic reticulum, and NADPH oxidase (NOX) in hepatocytes have profound and direct impacts on HSCs activation (17, 23, 24). In addition, the receptors for advanced glycation end-products (RAGEs), which is pattern recognition receptor, are highly expressed in HSCs. ROSs are also generated in AGE formation, and oxidized RAGE stimulates NOX1, which contributes to ROS production in HSCs (24). Other signals from hepatocytes, such as leptin and osteopontin, are also involved in mediating the transformation of HSCs into a profibrotic and inflammatory phenotype (17). It should be noted that the innate immune response mainly regulates aseptic inflammation triggered by metabolic stress (25, 26). The innate immune system activates the release of inflammatory cytokines and chemokines by sensing metabolic stress and many of them have shown to be important in the pathogenesis of fibrosis, such as IL-1β and IL-18, C-C chemokine ligand types 2 and types 5 (CCL2 and CCL5), together with C-C chemokine receptor type 2 and types 5 (CCR2 and CCR5), etc. (27–29).

Hepatic macrophages can also polarize toward a proinflammatory phenotype, and their TLR4 signaling facilitates the production of transforming growth factor-beta 1 (TGF-β1) in response to metabolic insults. Transforming growth factor-beta 1 coordinates with HSCs to accelerates liver fibrosis (30). Hepatic T cell population is also essential in NASH-associated inflammation or stellate cell activation. Maintaining a good amount of CD8^+^ tissue-resident memory T cells protected mice from fibrosis progression by predisposing activated HSCs to FasL-Fas-mediated apoptosis in a CCR5-dependent manner (31, 32). There is a large number of B cells in the liver, immune regulatory properties of HSCs promote the profibrogenic activity of B cells (33). Platelets are an essential cellular source of PDGFβ and TGFβ that activate HSCs and promote fibrosis in NASH. Extracellular signals from resident and inflammatory cells collectively modulate HSCs activation by stimulating autophagy, oxidative stress, endoplasmic reticulum stress, and retinol metabolism, thereby further modulating liver fibrosis (17). Since NAFLD is a component of metabolic syndrome that affects multiple organs, circulating factors, and signals from extrahepatic tissues and organs, such as the intestinal microbiome (34), adipose tissue, and skeletal muscle, it also influences liver fibrosis (17, 35). Especially, intestinal flora-derived pathogen-associated molecular patterns and danger-associated molecular patterns, as well as endotoxins, could directly promote fibrosis by signaling through innate immune receptors like TLR4 on HSCs (17). Different types of epigenetic modifications, including DNA methylation, histone covalent modifications, and the expression of some non-coding RNAs (such as miR-29a and miR-590-5p), were found to play essential roles in regulating HSCs activation during the progression of NAFLD to NASH (36, 37).

Given the development of liver fibrosis as a result of interactions between various hepatic cell types under metabolic stress and the mediation of intercellular communication by secreted mediators or circulating mediators, which regulate lipid toxicity, inflammation, apoptosis, extracellular matrix formation, and fibrosis, the discovery of molecular targets and the development of novel pharmacological strategies covers all these aspects.

## Liver Fibrosis Driven by Non-Esterified Fatty Acid-Derived Lipid Synthesis

### Glucagon-Like Peptide-1 and GLP-1 Agonists

Glucagon-like peptide-1 (GLP-1) is a pleiotropic peptide hormone excreted by intestinal L cells that enhances insulin secretion and improves glucose homeostasis, thereby reducing liver non-esterified fatty acid (NEFA) overload caused by triglyceride decomposition. In addition to its metabolic benefits, GLP-1 has been shown to delay gastric emptying and limit body weight, as well as inhibit inflammation and cell apoptosis (38). These features make GLP-1 receptor agonists well-suited for the treatment of MAFLD, which is characterized by metabolic disorders, and allow them to help reduce multiple upstream links of HSCs activation, such as IR, lipid toxicity, and metabolic inflammation. Animal studies have shown that GLP-1 receptor agonists alleviate hepatic steatosis and inflammation, and play an antifibrotic role by improving HSCs phenotypes (12).

Liraglutide is a long-acting GLP-1 receptor agonist that reduces body weight in NAFLD patients. In a phase 2 study (NCT01237119), NAFLD patients who received subcutaneous injections of Liraglutide achieved histological improvement with attenuated fibrosis progression (39). Adverse effects of Liraglutide mainly consist of mild to moderate gastrointestinal reactions. Another GLP-1 receptor agonist in clinical application is Exenatide, which yields better improvement in the noninvasive Fibrosis 4 index and greater benefit in terms of body weight and liver enzymes than insulin Glargine in NAFLD patients with type 2 diabetes (NCT02303730) (40). A new-generation GLP-1 receptor agonist, Semaglutide, also improved steatohepatitis in a phase 2 trial (NCT02970942). It should be noted that although the administration of Semaglutide improved the fibrosis stage in nearly half of the patients, the excessive fibrosis regression rate in the placebo group made the difference between the groups non-significant (43 vs. 33%, *P* = 0.48) (41). Cotadutide is a dual receptor agonist of GLP-1 and Glucagon. Preclinical evidence has demonstrated that it is more effective than Liraglutide in improving liver fibrosis in NASH models (42). The current phase 2 study of NAFLD with compensatory fibrosis in patients with obesity (NCT04019561) completed the data collection of primary outcomes. Tirzepatide, a dual receptor agonist of GLP-1 and glucose-dependent insulinotropic polypeptide, also reported an improvement in fibrosis biomarkers in NAFLD patients with type 2 diabetes (NCT03131687) (43). Although it has only been approved by the US-FDA for the treatment of type 2 diabetes at present, a wide variety of GLP-1 receptor agonists have become attractive candidate drugs for the treatment of MAFLD due to their metabolic benefits.

## Liver Fibrosis Driven by *De Novo* Lipogenesis-Derived Lipid Synthesis

### Acetyl-CoA Carboxylase and ACC Inhibitors

*De Novo* lipogenesis (DNL) is a pivotal step in liver fatty acid metabolism and plays a major role in triglyceride accumulation in hepatocytes (44). Acetyl-CoA carboxylase (ACC) is a crucial enzyme in DNL regulation that catalyzes the rate-limiting step of acetyl-CoA to malonyl-CoA conversion and modulates mitochondrial fatty acid oxidation. Therefore, ACC is an attractive therapeutic target for restoring the balance of hepatic fatty acid metabolism (44). An animal study confirmed that inhibiting ACC reduced lipid toxicity in hepatocytes by lessening DNL. This mechanism resulted in the direct suppression of HSCs activation and impairment of HSCs profibrogenic activity, thereby reducing liver fibrosis in a rat model (45).

Firsocostat (also known as GS-0976 and NDI-010976) is a small-molecule allosteric inhibitor of ACC in the liver that significantly inhibited hepatic DNL in a metabolically overburdened population in a dose-dependent manner (46). Treatment with 20 mg of Firsocostat daily for 12 weeks reduced hepatic steatosis and fibrosis marker levels in patients with NASH (NCT02856555) (47). In order to explore more suitable treatment regimens for NAFLD patients with advanced liver fibrosis, the ATLAS phase 2b study (NCT03449446) focused on the improvement of F3–F4 fibrosis after 48 weeks of treatment with three agents alone or in combination (48). The combination of Firsocostat and either of the other two drugs allowed more patients to achieve the primary endpoint of fibrosis improvement ≥ 1 stage than the monotherapy regimen, although this benefit did not have a significant advantage over placebo. The combination of 20 mg of Firsocostat and 30 mg of Cilofexor transformed the fibrosis pattern in the biopsy area into ≤F2 and led to significant improvements in NASH activity, the enhanced liver fibrosis score and the liver stiffness as determined by transient elastography, revealing a potential antifibrotic effect (48). Most patients treated with Firsocostat experienced a remarkable increase in serum triglyceride levels. This asymptomatic hypertriglyceridemia subsided spontaneously in some patients and could partially be resolved by treatment with fish oil or fibrates (47, 48). Hypertriglyceridemia may be the result of decreased polyunsaturated fatty acids produced by malonyl-CoA, which makes the expression of sterol regulatory element-binding protein 1 increase in a compensatory manner, resulting in increased very-low-density lipoprotein secretion in the liver and peripheral triglyceride accumulation (44). In addition, Firsocostat in combination with Cilofexor increased the total cholesterol level and decreased the high-density lipoprotein cholesterol level in the ATLAS study (48). In consequence, further research on the long-term effects of Firsocostat on the cardiovascular system is needed.

PF-05221304 is another oral liver-directed ACC inhibitor that significantly improved the hepatic steatosis, fibrosis, and inflammation induced by metabolic stress in both human-derived *in vitro* systems and rodent models (49). An ongoing phase 2 study (NCT04321031) is evaluating whether it has a beneficial effect on fibrosis as assessed by liver biopsy in NASH patients.

### ATP-Citrate Lyase and ACLY Inhibitors

ATP-citrate lyase (ACLY) is a pivotal lipogenic enzyme positioned at the coupling between glycocatabolism and lipid anabolism, and serves as a metabolic checkpoint for detecting excessive nutrients (12, 50). ATP-citrate lyase catalyzes mitochondrial-derived citrate into cytoplasmic oxaloacetate and acetyl-CoA, which in turn promotes the synthesis of fatty acids and cholesterol, and the acetylation of proteins (50). Studies have shown that the expression of ACLY is increased in liver samples from NAFLD patients (51). The increase of ACLY in hepatocytes may be due to the impairment of an E3 ligase-drived ubiquitination-dependent degradation of ACLY during metabolic stress (52). ATP-citrate lyase is involved in inflammatory and IL-4-induced macrophage polarization and activates the transcription factor Stat6 to induce coordinated fibrosis and tissue remodeling (50, 53). ATP-citrate lyase activity inhibition and gene silencing help prevent hepatic steatosis, reduce oxidative stress and prostaglandin E2 inflammatory mediators, thereby indirectly contribute to the improvement of fibrosis in metabolism-induced liver disease (12, 54, 55). Bempedoic acid (ETC-1002) is a first-class, prodrug-based direct competitive inhibitor of ACLY, which reduces ACLY activity and hepatic lipids in rodents (55, 56). Analysis of its phase 2 and 3 clinical studies demonstrated that bempedoic acid has good safety and favorable effects on lipid profiles and inflammation represented by high-sensitivity C-reactive protein (57). This evidence suggests that ACLY may serve as a new therapeutic target for regulating metabolism and MAFLD-related fibrosis. Further clinical trials are needed to evaluate the safety and efficacy of bempedoic acid in improving the primary outcomes of metabolic overload-induced liver disease and its fibrosis.

### Fatty Acid Synthase and FASN Inhibitors

As a member of the lipogenic enzymatic cascade, fatty acid synthase (FASN) is a core modulator of hepatic DNL that catalyzes the synthesis of palmitate from acetyl-CoA and malonyl-CoA (58, 59). The powerful rate-limiting capacity of FASN for lipogenesis makes it a promising target for the treatment of liver disease caused by metabolic stress. The inhibition of FASN reduced hepatic DNL and improved liver steatosis in animal models and in patients with NAFLD (58–60). TVB-2640 is a novel FASN inhibitor that significantly reduces liver DNL and hepatic fat in obese subjects after 10 days' treatment (NCT02948569) (61). Given that the inhibition of hepatic DNL decreases intrahepatic fat accumulation, inflammation and fibrosis, a FASCINATE-1 (NCT03938246) phase 2a study focused on the efficacy of 25 mg or 50 mg of TVB-2640 in NASH patients (62). After 12 weeks of treatment, TVB-2640 reduced the liver fat and inflammatory biomarkers levels in a dose-dependent manner. More encouragingly, TVB-2640 produced significant improvements for several serum markers of fibrosis in NASH patients after such a short-term treatment (62). The benefit of TVB-2640 on fibrosis may be a result of the reduction in the DNL-triggered activation of HSCs or from inhibiting the indirect effect of lipotoxicity-mediated fibrosis (62). Encouraged by the success of the pilot study, a 52-week long-term study FASCINATE-2 (NCT04906421) was initiated with a higher drug dose (50 or 75 mg) and is recruiting NASH patients with F2–F3 liver fibrosis.

### Stearoyl-CoA Desaturase 1 and SCD1 Inhibitors

Stearoyl-CoA desaturase 1 (SCD1) is also a key enzyme for hepatic lipid anabolism that catalyzes the rate-limiting step of converting saturated fatty acids into monounsaturated fatty acids (63). Studies have indicated that SCD1 is overexpressed in activated HSCs, and is involved in diet-induced steatohepatitis and fibrosis by regulating Wnt signaling (64, 65). Decreasing SCD1 expression through genetic disruption or pharmacological inhibition reduced HSCs activation and alleviated liver fibrosis and steatohepatitis in murine models (66, 67). Arachidyl-amido cholanoic acid (Aramchol) downregulated SCD1 to inhibit DNL in the liver, reduce steatosis and inflammation, and reverse fibrosis in mice (66). In a phase 2a clinical trial (NCT01094158), 300 mg of Aramchol daily for 3 months dramatically reduced the liver fat content in NAFLD patients without significant adverse effects (67). Because the parameters in the above study, such as liver enzymes and insulin sensitivity did not improve, and the efficacy of the 300 mg was better than that of 100 mg, the ARREST phase 2b trial (NCT02279524) carried out a further study with doses of 400 and 600 mg. At the end of the 1-year treatment, Aramchol demonstrated dose-dependent benefits in reducing hepatic steatosis and fibrosis with good tolerability (63). Therefore, the ARMOR phase 3 trial (NCT04104321) was launched to evaluate the efficacy and safety of 300 mg of Aramchol twice a day in NASH patients with stage 2–3 fibrosis and metabolic disorders. The primary endpoint is fibrosis improvement for stage 1 or above and NASH regression, and the trial is currently in the recruitment stage (68).

## Liver Fibrosis Driven By Dietary Intake-Derived Lipid Synthesis

### Fanitol X Receptor and FXR Agonists

Fanitol X receptor (FXR) is a nuclear receptor widely expressed in the liver and small intestinal mucosa that plays an essential role in the sensation of bile acid signals. Fanitol X receptor senses bile acid signals and regulates their secretion by negative feedback, resulting in decreased intestinal lipid absorption, downregulation of the expression of key lipogenic genes in the liver, and reduced hepatic lipid levels in the end (69). The beneficial effects of the ligation of FXR with bile acids on metabolism also include promoting fatty acid oxidation in the liver, regulating glycogenolysis and gluconeogenesis, as well as restoring insulin sensitivity in muscle and adipose tissue (12). These processes help to reduce toxic lipid production and suppress HSCs activation. The activation of FXR in HSCs has been confirmed to reduce extracellular matrix production and weaken the response of HSCs to profibrotic signals such as TGF-β, thus playing a protective role in fibrosis (70). In patients with fatty liver disease, the expression of hepatic FXR was negatively correlated with disease severity. In a rodent model, the deletion of FXR in the liver acerbates metabolic stress-induced liver steatosis, inflammation and fibrosis (12). Therefore, agonists of FXR are emerging as promising therapeutic agents for treating metabolic stress-induced liver fibrosis.

Obeticholic acid (OCA) is a semisynthetic FXR agonist that is more than 100-fold more potent than the endogenous ligand chenodeoxycholic acid (12). There is evidence from a number of clinical trials and animal studies showing that OCA has a promising effect on improving fibrosis due to NASH (71–73). Animal studies have indicated that OCA reduces metabolic stress-induced liver fibrosis and lipid infiltration and effectively improves systemic IR in obese and diabetic mice (12, 74). In the FLINT phase 2 clinical trial (NCT01265498), liver fibrosis and disease histological features were significantly improved in NASH patients who received 25 mg of OCA daily compared to those who received placebo (72). The benefits of OCA on NASH-induced liver fibrosis were also identified in interim analysis results published in the ongoing REGENERATE phase 3 trial (NCT02548351). Taking 25 mg of OCA daily improved liver fibrosis in nearly a quarter of NASH patients with stage 2–3 fibrosis, which is approximately twice the proportion observed in the control group (73). Twenty-five milligrams of OCA also outperformed placebo in reducing the NAFLD activity score (NAS). Regrettably, the expected endpoint of NASH regression was not achieved after 18 months of OCA treatment. The limitations of OCA treatment include not only mild to moderate dose-dependent pruritus but also more worrisome dyslipidemia, characterized by elevated low-density lipoprotein cholesterol (LDL-c) levels during treatment, which may pose an additional risk of atherosclerosis in NASH patients who are already overweight or suffering from type 2 diabetes (73). Overall, the treatment benefits on mid-term histological endpoint of the REGENERATE study are still uncertain, and the benefits do not significantly outweigh the potential risks. Therefore, in June 2020, the US-FDA rejected the pharmaceutical company Intercept's application for approval of OCA in treating fibrosis due to NASH, recommending that Intercept provide more interim data from REGENERATE and maintain long-term studies on the benefit-risk ratio of OCA. Currently, the efficacy and safety of OCA therapy in patients with compensated cirrhosis due to NASH are being evaluated in a phase 3 clinical trial (NCT03439254). Considering that FXR activation influences plasma lipoprotein concentrations, the combination of OCA and statins was considered and tested in the CONTROL phase 2 study (NCT02633956). After 16 weeks of treatment, OCA-induced increases in LDL-c in patients with NASH were mitigated with atorvastatin. This combination was generally safe and well-tolerated (75).

In addition to OCA, several FXR agonists have been examined in clinical trials or proven to be effective in animal studies. Cilofexor (formerly GS-9674), a small-molecule non-steroidal agonist of FXR, significantly reduced hepatic steatosis, liver biochemical marker, and serum bile acid levels, but did not improve liver fibrosis or stiffness after 24 weeks of treatment (NCT02854605) (76). However, as previously mentioned, it has preferable antifibrotic potential in combination with the ACC inhibitor Firsocostat (NCT03449446). A novel FXR agonist, EDP-305, also demonstrated a potent effect in terms of reducing liver fibrosis triggered by metabolic stress, bile duct ligation, and methionine-choline deficient (MCD) diet in rodent models (77, 78). Similar compounds, e.g., Tropifexor (LJN452) (NCT03517540 and NCT04065841), Nidufexor (LMB763), PX-104, EYP001, and TERN-101, are under investigation (79). In summary, FXR signaling decreases in patients with metabolic overload-induced fatty liver disease, especially in the fibrotic stages. Restoring FXR expression using a FXR agonist demonstrates promising therapeutic potential for treating fatty liver disease and fibrosis. Currently, there are side effects that limit the application of this strategy in clinical practice. Thus, the development of more precise drug targets or combination therapies is warranted.

Fibroblast growth factor 19 (FGF19), secreted by the intestines during feeding, is a signaling hormone downstream of FXR activation and is currently under clinical investigation for the potential treatment of NASH and its associated fibrosis (80). Fibroblast growth factor 19 (mouse ortholog FGF15) promotes NEFA oxidation in mitochondrial and glycogen synthesis via its interaction with FGF receptors (FGFR) in hepatocytes and thus suppresses metabolic stress-induced signaling activation in HSCs and liver fibrosis (80, 81). In addition, FGF19 plays a crucial role in the regulation of systemic glucose and lipid metabolism, as well as maintaining energy homeostasis (81). However, efforts involving the FGF19-FGFR pathway have encountered setbacks. Carcinogenicity were observed in mice treated with FGF19 (81, 82). To overcome these barriers, alternative approaches and molecules need to be developed. Aldafermin (alias NGM282) is an engineered non-carcinogenic FGF19 analog that maintains a key region of the protein involved in receptor interactions and signaling modulation but does not activate the FGF19-STAT3 carcinogenic pathway (82, 83). A 5-aminoacid deletion (P24–S28) coupled with the substitution of three amino acids at crucial positions (Ala30Ser, Gly31Ser, and His33Leu) within the amino terminus of Aldafermin enables biased FGFR4 signaling; thus, Aldafermin retains the ability to potently repress CYP7A1 expression (82). The first phase 2 trial (NCT02443116) assessing the safety and efficacy of Aldafermin for the treatment of NASH revealed that 12 weeks of Aldafermin treatment rapidly decreased liver fat content measured by MRI-proton density fat fraction (MRI-PDFF), and non-invasive markers of inflammation and fibrosis (84). Because the histological features of NASH were not assessed at the end of this study, an open-label trial of Aldafermin with histological endpoints in patients with NASH was conducted (NCT02443116). Consistently, this study confirmed that Aldafermin improved the NAS and fibrosis score of NASH patients after 12 weeks of treatment (85). Due to the encouraging success in trials of 12 weeks of Aldafermin treatment, the efficacy and safety of 24 weeks of treatment were further evaluated in patients with biopsy-proven NASH (NCT02443116). In this trial, Aldafermin reduced liver fat and produced a trend toward fibrosis improvement, and few adverse events were reported (86). To further evaluate the benefits of Aldafermin on liver fibrosis, two multicenter randomized controlled trials (RCTs) of Aldafermin in subjects with F2/F3 fibrosis (NCT03912532) or compensated cirrhosis (NCT04210245) are underway. It is worth mentioning that Aldafermin modulates CYP7A1-mediated bile acid homeostasis and may lead to an increase in serum cholesterol. An appropriate combination with statins may counteract Aldafermin-induced side effects on the lipid profile (NCT02443116) (87). Although no liver tumors were observed in multiple animal models after prolonged exposure to Aldafermin (82), trials of longer duration are still warranted for further safety evaluation.

Fibroblast growth factor 21 (FGF21) demonstrates large functional overlap with FGF19 in terms of regulating energy homeostasis and metabolism (80, 81). Unlike FGF19, FGF21 is produced in the liver during fasting and in response to elevated NEFA levels. The increased level of FGF21 in plasma has a negative-feedback effect on lipolysis in peripheral tissue (81). Moreover, FGF21 facilitates glucose and lipid uptake and adipogenesis in adipose and muscle tissue, which prevent ectopic lipid accumulation in the liver (80). Fibroblast growth factor 21 has shown great therapeutic potential as a treatment for NASH, but it has poor pharmacokinetic and biophysical properties (88). Numerous FGF21 analogs have been synthesized and developed for the treatment of metabolic diseases. Pegbelfermin (BMS-986036), a PEGylated human FGF21 analog, was tested in patients with NASH in a phase 2a clinical trial (NCT02413372). The administration of Pegbelfermin for 16 weeks significantly reduced the hepatic fat fraction as measured by MRI-PDFF by over 10%, and deceased liver fibrosis biomarkers, e.g., N-terminal type III collagen propeptide (pro-C3) (89). Further research using liver biopsies to assess the effects of 24 weeks of Pegbelfermin treatment on patients with histologically confirmed NASH with stage 3 liver fibrosis (FALCON 1; NCT03486899) or compensated cirrhosis (FALCON 2; NCT03486912) was initiated in 2018 (90). Efruxifermin is an engineered fusion protein formed by linking human IgG1 Fc to modified FGF21, and it has a balanced and long-lasting agonistic effect on FGFR1c, 2c, and 3c (91, 92). In the 16-week phase 2a BALANCED study (NCT03976401), Efruxifermin resulted in a significant reduction in the liver fat fraction as measured by MRI (92). This improvement in hepatic steatosis was accompanied by a reduction in biomarkers of fibrosis and the enhanced liver fibrosis scores. More importantly, 55% of patients achieved stage 1 or greater fibrosis improvement, and half of these patients even met all the exploratory endpoints (92). The major adverse effects of FGF21 analogs is mild gastrointestinal reactions. The promising efficacy and mild side effects of FGF21 analogs demonstrate their potential as treatments for NASH.

## Liver Fibrosis Driven By Multiple Metabolic Pathway-Derived Lipid Synthesis

### Peroxisome Proliferator-Activated Receptors and PPAR Agonists

Peroxisome proliferator-activated receptors (PPAR), a superfamily of nuclear hormone receptors, are extensively involved in the regulation of metabolic homeostasis and inflammatory response in the liver (63). The distribution and functions of the three PPAR hypotypes, α, δ, and γ, are not identical. PPARα is mainly located on liver cells. After activation, it promotes the oxidation of fatty acids in the liver and enhances the expression of superoxide dismutase and catalase to protect liver cells from oxidative stress-induced damage (12). The expression of PPARδ is more extensive and serves various functions, such as inhibiting inflammation, enhancing NEFA oxidation, suppressing adipogenesis, and regulating the immune system (12). PPARα and PPARδ play an important role in suppressing liver fibrosis by inhibiting liver steatosis and inflammation (93). PPARγ is mainly expressed in adipocytes and pancreatic β cells and is able to accelerate the differentiation and storage capacity of adipocytes and regulate glucose metabolism (12, 94). Importantly, PPARγ can be activated by various ligands, such as fatty acids and thiazolidinedione, to inhibit HSCs proliferation and improve liver fibrosis (93). Research-based evidence suggests that PPARγ mediates the effect of liver-protective docosahexaenoic acid in ameliorating liver fibrosis by inducing cell cycle arrest and apoptosis in HSCs (95). In summary, some agonists targeting PPAR may have promise for the treatment of liver fibrosis.

Pirfenidone is an oral PPARα agonist with antisteatogenic and antifibrotic effects that is currently approved for the treatment of idiopathic pulmonary fibrosis (96). To evaluate its value for application in treating advanced liver fibrosis, the PROMETEO phase 2 study (NCT04099407) applied a sustained-release formulation with less potential toxicity to liver metabolism and a longer-lasting plasma concentration (97). The ratio of stage 3 to stage 4 fibrosis in the study population was approximately 1:3, and nearly half of the patients had advanced fibrosis due to NAFLD. Taking 600 mg of Pirfenidone twice a day improved fibrosis in 35% of patients after 12 months and reduced liver enzyme levels in nearly half of the patients. Moreover, the serum TGF-β1 level was lower, and the quality of life appraised by the Euro-QoL scale was better after Pirfenidone treatment. As the serum Pirfenidone concentration was higher in patients with fibrosis regression than in patients with fibrosis progression, Pirfenidone was associated with a better antifibrotic effect (97). The PPARγ agonist Pioglitazone, a first-generation thiazolidinedione agent, has been shown to improve the fibrosis score in NASH patients without diabetes (NCT00994682) (98). However, for patients with diabetes, the combination of Pioglitazone and vitamin E did not improve liver fibrosis, although this regimen was superior to vitamin E alone or placebo in terms of steatohepatitis resolution (NCT01002547) (99). Due to side effects, such as fluid retention, osteoporotic fracture or hypoglycemia, Pioglitazone may increase the overall risk of patients with metabolic disorders, and second-generation PPARγ agonists have been developed and tested in clinical trials. Novel agents, such as MSDC-0602K, limited the common side effects of Pioglitazone. However, MSDC-0602K failed to improve liver histological features in NASH patients with stage 1–3 fibrosis in a 52-week phase 2b trial (NCT02784444) (100).

Agonists that act on multiple PPAR hypotypes at the same time seem to be more effective than those that only act on one PPAR hypotype. Elafibranor (formerly GFT505) is a dual-pathway agonist that acts on both PPARα and PPARδ. It has been researched in a phase 2b study of NAFLD patients without cirrhosis (NCT01694849). In a *post-hoc* analysis aimed at the degree of steatohepatitis resolution, the researchers found that 120 mg of Elafibranor daily had a better effect on disease activity in the population with a NAS ≥4. More importantly, patients who achieved the primary outcome are often accompanied by a reduction in the degree of liver fibrosis (101). Another dual-path agonist, Saroglitazar, targets PPARα and PPARγ and has shown beneficial effects on serum lipid levels and liver biochemical parameters in patients with NAFLD (NCT03061721) (102, 103). It leads to improvement in non-invasive-assessed liver fibrosis parameters in NAFLD patients with diabetic dyslipidemia (104). It may produce antifibrotic effects by reducing oxidative stress and the production of lipotoxic substances, as well as inhibiting leptin signaling. The efficacy and safety of this drug in NAFLD patients with advanced fibrosis are still under evaluation (NCT04469920). Lanifibranor (IVA337), a pan-PPAR agonist that acts on all three receptor subtypes, causes fibrosis regression and ameliorates HSCs-related phenotypes in preclinical models of advanced chronic liver disease (105). In the phase 2b study NCT03008070 completed just a few months ago, Lanifibranor reached the steatosis active fibrosis score endpoint and demonstrated histological fibrosis improvement, with good tolerability. In the forthcoming multicenter phase 3 study NCT04849728, Lanifibranor will be further evaluated in adult patients with non-cirrhotic NAFLD and stage 2/3 liver fibrosis. In general, the benefits of poly/pan-PPAR agonists for liver fibrosis appear to be better than those of single-subtype agonists. We still need a lot of clinical evidence to highlight the direction for the application of such drugs.

### Thyroid Hormone Receptor-β and THR-β Agonists

As with the PPAR family, thyroid hormones widely participate in the regulation of lipid and glucose metabolism. Thyroid hormone receptor (THR)-β is mainly expressed in the liver and specifically enhances the oxidative utilization of hepatic fat and cholesterol metabolism (12). Preclinical studies revealed that the specific activation of THR-β reduces hepatic steatosis and fibrosis and improves insulin sensitivity and hepatocyte injury (106). There are two selective THR-β agonists presently under clinical development that can optimize the liver benefits while avoiding the adverse cardiac and skeletal effects of activating THR-α.

Resmetirom (MGL-3196) is a liver-directed THR-β agonist. In a phase 2 study (NCT02912260), the oral administration of 80 mg of Resmetirom daily for 36 weeks improved fibrosis activity markers in NAFLD patients with stage 1–3 fibrosis and caused the resolution of steatohepatitis in nearly 30% of patients, who also showed an improvement in the liver fibrosis stage compared with those treated with placebo (107). To obtain more data on the safety and efficacy of Resmetirom in non-invasive assessments, an open-label extension study (NCT02912260) was conducted in 31 patients from the aforementioned study with sustained mild to significantly increased liver enzyme levels. A reduction in fibrosis markers such as pro-C3 and liver stiffness assessed by transient elastography was observed after 36 weeks of Resmetirom treatment (108). Unlike the aforementioned OCA or Aldafermin, Resmetirom resulted in reduced levels of multiple lipids that carry the risk of atherosclerosis, such as LDL-c and triglycerides (108). In addition, Resmetirom was well-tolerated, and the main adverse effects were transient mild diarrhea or nausea (107, 108). The considerable efficacy and safety support the ongoing phase 3 clinical study (NCT03900429) to explore the efficacy for NAFLD patients with F2–F3 fibrosis. Another selective THR-β agonist is VK2809 (formerly called MB07344), which significantly improved liver fat content in NAFLD patients with hyperlipidemia (NCT02927184); the publication of the study result is expected. A 52-week phase 2b study, VOYAGE, is currently being conducted in NAFLD patients with stage F1–F3 fibrosis to investigate the benefits of 1.0, 2.5, 5.0, and 10 mg of VK2809 compared with placebo on liver fat content, fibrosis, and histopathology (NCT04173065). Compared with VK2809, Resmetirom has more clinical evidence to support its therapeutic potential in NASH.

## Liver Fibrosis Driven By Cellular Stress and Apoptosis

### Vitamin E

Excessive fatty acids and subsequent mitochondrial dysfunction lead to ROS production, which plays a crucial role in NASH and advanced fibrosis (17, 24, 109, 110). Vitamin E, as a major fat-soluble chain-scission antioxidant, prevents plasma lipid and low-density lipoprotein peroxidation and protects the structural integrity of cells from damage caused by lipid peroxidation and oxygen-free radicals (110, 111). In addition to its powerful antioxidant effects, vitamin E also induces adiponectin expression, reduces inflammatory signaling, and regulates macrophage polarization, making it a potential treatment option for suppressing oxidative stress and metabolism-related liver diseases (111, 112). In the metabolic stress-induced NASH model in mice, vitamin E reduces oxidative stress, improves hepatic fibrosis and HSCs activation, and alleviates hyperinsulinemia. The antifibrotic properties may be due to the inhibition of TGF-β expression after the downregulation of ROS production, thereby reducing the activation of HSCs (112). A clinical study indicated that using 800 IU of vitamin E per day significantly improved transplant-free survival and liver decompensation in NASH patients with stage F3–F4 fibrosis (113). However, there is still a lack of direct and conclusive evidence of the beneficial effect of vitamin E on NASH-induced liver fibrosis. Vitamin E did not cause significant regression of liver biopsy-proven fibrosis in RCTs (NCT00063622, NCT00063635, and NCT01002547), although it induced varying degrees of improvement in NASH histology (99, 114, 115). In general, the liver benefits of vitamin E alone were more embodied in the reduction of oxidative stress marker levels and improvement of liver function and the NAS (99, 111, 116). It must be emphasized that there is evidence suggesting that dietary vitamin E supplementation might increase cancer risk (NCT00006392) and mortality in the healthy population (117, 118). However, meta-analyses from recent years have shown that the adverse effects of vitamin E supplementation on all-cause mortality or cancer risk are not significant, supporting dietary intake of this natural antioxidant (119, 120). Therefore, hepatologists may be inclined to use vitamin E in combination with other reagents in the study of NAFLD/MAFLD.

### Caspase Inhibitors

Chronic liver injury induced by excessive toxic lipid leads to increased hepatocyte apoptosis, which is an important feature of NASH (121, 122). Apoptotic hepatocytes activate immune cells and HSCs, thereby promoting liver fibrosis and cirrhosis (122, 123). Caspases, a family of cysteine proteases, play a central role in the progression of NAFLD/NASH due to their role in the regulation of liver apoptosis and inflammation (124). Caspase inhibitors have been studied and tested as therapeutic agents for NASH (122). Emricasan (IDN-6556) is an irreversible pan-caspase inhibitor that ameliorates apoptosis and liver fibrosis in NASH mouse models (125). In a short-term clinical study (NCT02077374), Emricasan suppressed caspase activation and liver enzyme levels in patients with NASH after 4 weeks of treatment, with decent safety and tolerance (126). Moreover, 3 months of treatment with Emricasan improved liver function more in patients with cirrhosis caused by NASH than in those with cirrhosis caused by viral hepatitis or alcoholic liver disease and showed a potential beneficial effect on portal hypertension (NCT02230670) (127). However, Emricasan did not show a significant beneficial effect on fibrosis regression in RCTs with a larger sample of NASH patients. In NASH patients with stage F1–F3 fibrosis (NCT02686762), usage of Emricasan at 10 or 100 mg daily for 72 weeks failed to improve liver fibrosis or lead to NASH resolution. Moreover, fibrosis and hepatocyte ballooning were aggravated in the Emricasan group (128). In another study conducted in NASH patients with compensatory cirrhosis (NCT02960204), Emricasan at 10, 50, or 100 mg daily did not cause an improvement in clinical outcomes (129). In NASH patients with decompensated cirrhosis (NCT03205345), administering 10 or 50 mg of Emricasan daily also did not reduce the amount of decompensation events or improve liver function after 48 weeks (130). These three studies indicated a robust effect of Emricasan on caspase activity inhibition and a good safety, but none demonstrated a significant therapeutic benefit. This may be due to excessive inhibition of apoptosis activating alternative forms of cell death, such as necroptosis and pyroptosis (124, 128). It is also possible that cirrhosis leads to many pathological changes, such as reduced number of functional hepatocytes, decreased hepatic blood flow and transporter protein expression, resulting in unsatisfactory drug bioavailability. Therefore, a daily dose of 50–100 mg may be insufficient for patients with cirrhosis (129, 130). Although none of these studies achieved the primary endpoint, they provided valuable reference data and ideas for design optimization in future clinical research on NASH-associated fibrosis. In a recent study in an HCV patient treated with liver transplantation, 24 months of Emricasan therapy showed a beneficial effect on moderate liver fibrosis. Although the pathogenesis of HCV-related fibrosis differs from that in NASH, this positive result inspires the initiation of treatment in NASH patients with moderate fibrosis (131).

## Liver Fibrosis Driven by the Innate Immune System and Inflammation

The liver consists of a network of innate immune cells, which collectively form the first line of defense against invading organisms and toxins (132). Under excessive metabolic stress, the hepatic innate immune system is over-activated to further trigger hepatic cell injury and liver fibrosis (22, 25, 133, 134). There are a number of molecular targets that function as hubs controlling the inflammatory signaling flow in the progression of fatty liver disease, and they have been emerging as targets in the development of drugs for the treatment of MAFLD/NAFLD and fibrosis (48, 135–140).

### Apoptosis Signal-Regulating Kinase 1 and ASK1 Inhibitor

Studies have revealed that apoptosis signal-regulating kinase 1 (ASK1), a member of the mitogen-activated protein kinase kinase kinase (MAP3K) family, is hyperactivated in the liver of NASH patients. Upon receiving metabolic stress signals, ASK1 activates the downstream c-Jun N-terminal kinase (JNK) 1/2-mitogen-activated protein kinase 14 (p38) signaling cascade to trigger hepatic inflammation and fibrosis during the development of MAFLD (135, 136). Apoptosis signal-regulating kinase 1 functions as a molecular hub controlling cellular signal transduction in NASH. It has been considered an essential target for the development of drugs for NASH.

A number of clinical trials evaluating the efficacy of ASK1 inhibitors against NASH have been performed. There have been two large phase 3 studies in patients with NASH and advanced fibrosis. They compared the effect of the ASK-1 inhibitor Selonsertib (GS-4997) with that of placebo in ~1,700 patients with NASH and bridging fibrosis (F3, STELLAR-3) or compensated cirrhosis (F4, STELLAR-4) (NCT03053050 and NCT03053063). Although Selonsertib successfully suppressed the expression of hepatic phospho-p38, it did not significantly improve liver fibrosis on liver biopsy (137, 138). There are several explanations for the failure of these large trials. First, there are a number of signaling pathways involved in the pathogenesis of NASH, particularly in the advanced stages, which suggests that combination therapy may be required in the treatment of NASH. In a phase 2 clinical trial, 72 patients with NASH and stage F2–F3 fibrosis were treated with either 6 or 18 mg of GS-4997 orally once daily alone or in combination with a once-weekly injection of 125 mg of Simtuzumab (a humanized monoclonal antibody directed against lysyl oxidase-like molecule 2) for 24 weeks (NCT02466516). Reduced liver hardness on MRI elastography, reduced collagen content and lobular inflammation on liver biopsy, and improved serum markers of apoptosis and necrosis all suggested improvement in liver fibrosis. The assessed results showed that the proportion of patients with a reduction of fibrosis of at least one stage at week 24 was 20% in the Simtuzumab -alone group (2 of 10; 95% confidence interval (CI), 3–56), 30% in the 6-mg Selonsertib group (8 of 27; 95% CI, 14–50), and 43% in the 18-mg Selonsertib group (13 of 30; 95% CI, 26–63). The changes in fibrosis stage were correlated with the changes in hepatic collagen content (*r* = 0.54, *P* < 0.001). The median percent change in the morphometric collagen content of patients who were treated with Simtuzumab alone was 2.1%, while that of patients treated with 6 and 18 mg of Selonsertib was −8.2 and −8.7%, respectively. In summary, compared with patients treated with Simtuzumab alone, patients treated with Selonsertib showed a higher rate of fibrosis improvement and a lower rate of fibrosis progression. These findings suggest that Selonsertib combined with Simtuzumab may reduce liver fibrosis in patients with NASH and stage 2–3 fibrosis (139).

Another study was performed to evaluate the safety and efficacy of Selonsertib, Firsocostat, Cilofexor, and combinations in participants with bridging fibrosis or compensated cirrhosis due to NASH (NCT03449446). In this study, 392 patients with bridging fibrosis or compensated cirrhosis due to NASH were randomized to receive 18 mg of Selonsertib, 20 mg of Firsocostat, or 30 mg of Cilofexor, alone or in two-drug combinations, once daily for 48 weeks. Histological parameter analysis showed that for the primary endpoint of an improvement in fibrosis of ≥ 1 stage without the worsening of NASH, the proportion of patients was 12% (4 of 33, *P* = 0.94) in the Firsocostat group, 12% (4 of 34, *P* = 0.96) in the Cilofexor group, 15% (11 of 71, *P* = 0.62) in the Firsocostat/Selonsertib group, 19% (13 of 68, *P* = 0.26) in the Cilofexor/Selonsertib group, and 21% (14 of 67, *P* = 0.17) in the Cilofexor/Firsocostat group. A higher response rate was observed in the combination groups than in the monotherapy groups, but the differences between the treatment and placebo arms did not reach statistical significance. However, patients treated with Cilofexor/Selonsertib (8%; *P* = 0.018 vs. placebo) were significantly less likely to progress to cirrhosis than those treated with placebo (41%). These results suggest that combination therapy with Selonsertib offers the possibility of fibrosis reversal in the long-term treatment of patients with advanced NASH and fibrosis (48). Additional studies are warranted to confirm the potential therapeutic effects of ASK1 inhibitors on liver fibrosis in NASH.

Since ASK1 plays essential roles in physiological function, modulating its activity via posttranslational modification could be a more appropriate strategy in the treatment of disease. Many ASK1-negative regulators have been reported to significantly inhibit the development of NASH-associated fibrosis in rodents and preclinical models (140–142). For instance, the disassociation of milk fat globule-epidermal growth factor-factor 8 from ASK1 accelerates its dimerization and phosphorylation in hepatocytes under metabolic stress, thus leading to liver steatosis and fibrosis (140, 141). The deubiquitinating enzyme tumor necrosis factor-alpha-induced protein 3 (TNFAIP3) directly interacts with and deubiquitinates ASK1 in hepatocytes and ameliorates metabolic stress-induced hepatic inflammation and fibrosis (144). Tumor necrosis factor receptor-associated factor 6 promotes the polyubiquitination of Lys6 connections and the activation of ASK1, in turn exacerbating inflammatory and fibrotic responses in the liver (143). A high-fat diet also induces the overexpression of hepatic E3 ligase Skp1-Cul1-F-box protein F-box/WD repeat-containing protein 5 (FBXW5), which is a key endogenous activator of ASK1 ubiquitination and activation, and small molecules that mimic FBXW5 (S1) and FBXW5 (S3) can block the ubiquitination of ASK1 in MAFLD (144). Future clinical trials could aim to these molecules that regulate the activity of ASK1 in the posttranslational modification process, which may lead to better therapeutic effects in the treatment of NASH.

### TGF-β-Activated Kinase 1 and TAK1 Inhibitors

TGF-β-activated kinase 1 (TAK1) is a member of the MAP3K family and is known as a central signalosome in the regulation of the inflammatory response (145). Conventionally, TAK1 is activated by proinflammatory cytokines and agonists of toll-like receptors to activate MAPK and NF-κB signaling pathways (146). There is accumulating evidence showed that metabolic stress also promotes TAK1 signalosome formation and activity in hepatocytes, which leads to the development of NAFLD and NASH (147). However, previous studies showed that the complete deletion of TAK1 expression also accelerates NASH progression, suggesting that maintenance of the normal enzymatic activity of TAK1 is also critical for sustaining homeostasis in metabolism and inflammation (148). Therefore, posttranslational modifications are essential in fine-tuning the activity of the TAK1 signalosome under such conditions. Recent studies have revealed endogenous molecules that are important in the regulation of TAK1 ubiquitination or phosphorylation without suppressing its physiological activity, which may serve as potential targets in the development of treatments for NASH. Evidences from mouse or preclinical non-human primate models showed that the deubiquitinating enzyme cylindromatosis, TNFAIP3-interacting protein 3, ubiquitin-specific protease (USP) 4, and USP18 mitigate liver steatosis, inflammation, and fibrosis by deubiquitinating metabolic stress-induced TAK1 ubiquitination and activation (149–153), while dual-specificity phosphatase 14 and regulator of G protein signaling 5 dephosphorylate TAK1, resulting in the reduced activation of TAK1 and its downstream signaling pathways (154, 155). Although these molecules show strong potency, their safety and efficacy required to be tested in prospective studies.

### Toll-Like Receptors and TLR4 Inhibitors

Due to the unique anatomical association of the liver with the intestine, the blood supply of the liver is enriched in microbial-associated molecular patterns (PAMPs) and nutrients. Thus, Toll-like receptors (TLRs) play an essential role in liver physiology and pathophysiology (156). Previous evidence has shown that TLRs are involved in the pathogenesis of NASH and liver fibrosis (157–159). Among these TLRs, the role of TLR4 has been the most extensively studied due to its importance in recognizing gut-derived endotoxin (160). The genetic deletion or pharmaceutical inhibition of TLR4 improved liver steatosis, inflammation, and fibrosis in response to a high-fat diet in mice and nonhuman primates (158). A small long-acting molecule, JKB-121, inhibits TLR4, which inhibits liver fibrosis by repressing the redox status and stellate cell activation in the liver (NCT02442687). Recently, a novel TLR4 antagonist, JKB-122, was developed and shown to be effective in reducing autoimmune hepatitis-associated liver necrosis and inflammation in animal models (161). A phase 2 study testing the efficacy of JKB-122 for 52 weeks in subjects with NASH with fibrosis was initiated in 2020 (NCT04255069).

### Vascular Adhesion Protein 1 and VAP-1 Inhibitor

Vascular adhesion protein 1 (VAP-1) is continuously expressed as a membrane-bound amine oxidase along the sinusoidal endothelium, which facilitates the accumulation of inflammatory cells into the inflamed environment in concert with other leukocyte adhesion molecules (162). The soluble form of VAP-1 (sVAP-1) is also found in the serum of healthy adults, and its expression is increased under inflammatory conditions and in metabolic disorders (163). Vascular adhesion protein 1 can modulate leukocyte migration in both its transmembranous and soluble forms. Studies have shown that hepatic VAP-1 and serum sVAP-1 expression is increased in patients with NAFLD compared with control individuals (164). In addition, VAP-1 plays an essential role in hepatic fibrosis due to a number of etiologies, such as NAFLD, HBV, and HCV (163–165). Mechanistically, VAP-1 directly affects stellate cells by enhancing the expression of profibrotic genes and promoting liver fibrosis (163). The VAP-1 mutant strain showed significant attenuations of liver inflammation and fibrosis in the MCD diet model (163). PXS-4728A is a selective and orally active VAP-1 inhibitor with potent efficacy observed in animal trials (166). In 2015, the data from a phase 1 clinical trial showed that PXS-4728A administered for 14 days at doses between 3 and 10 mg was safe and well-tolerated. The data suggest that low doses are effective in inducing persistent enzyme inhibition, but further clinical trials are needed to verify the effectiveness of the drug in treating NASH.

### C-C Chemokine Receptor and Ligand and CCR Antagonists

During NASH progression, C-C chemokine receptor type 2 (CCR2) and C-C chemokine receptor type 5 (CCR5), together with their respective ligands, C-C chemokine ligand types 2 (CCL2) and C-C chemokine ligand types 5 (CCL5), promote liver fibrosis by increasing immune cell aggregation and infiltration and amplifying the inflammatory response (167–170). Cenicriviroc is a dual CCR2 and CCR5 antagonist with significant antifibrotic and anti-inflammatory activity in models of fibrosis, such as the mouse peritonitis model, mouse diet-induced NASH model, and rat thioacetamide-induced liver fibrosis model (171). Recently, it has been explored in the treatment of liver fibrosis associated with NASH (172). The 2-year phase 2b CENTAUR study showed that Cenicriviroc treatment resulted in liver fibrosis improvement compared to placebo, with a greater effect on advanced fibrosis (172). Due to the success in the phase 2 trial, a phase 3 study examining the efficacy and safety of Cenicriviroc in the treatment of liver fibrosis in adults with NASH was initiated in 2017. Unfortunately, there was a lack of efficacy at the 12-month follow-up in terms of achieving an improvement in fibrosis of at least 1 stage with no worsening of steatohepatitis (NCT03028740). There are emerging studies investigating whether combination therapy provides superior clinical effectiveness in the treatment of NASH and fibrosis. A phase 2 clinical trial in NASH patients exploring the efficacy of a combination of Tropifexor (LJN452, an FXR agonist) and Cenicriviroc has been completed, and the results are pending publication (NCT03517540).

## Liver Fibrosis Driven by Other Mechanisms

### Lysyl Oxidase-Like 2 and LOXL2 Monoclonal Antibody

Lysyl oxidase-like 2 (LOXL2) is an extracellular copper-dependent enzyme that catalyzes the cross-linking of structural extracellular matrix components in fibrous organs, including the liver (173). The serum LOXL2 level was associated with the severity of liver fibrosis (174). In a preclinical model characterized by advanced fibrosis and portal hypertension, an anti-LOXL2 antibody decreased the portal pressure in Mdr2-knockout mice (175). However, the LOXL2 monoclonal antibody Simtuzumab failed to reduce the liver collagen content and fibrosis in NASH patients with advanced fibrosis and cirrhosis (NCT01672866 and NCT01672879) (176). These findings were also observed in a phase 2 trial of the combination of the ASK 1 inhibitor Selonsertib and Simtuzumab. The coadministration of Selonsertib and Simtuzumab did not provide additional benefits over Selonsertib therapy alone in patients with NASH and moderate to severe fibrosis (139). There are several potential factors explaining the failure of the Simtuzumab trial. First, LOXL2 may be the driver for NASH, and fibrosis or redundancy in other pathways may mediate collagen formation. Second, although Simtuzumab effectively binds LOXL2, the dose and frequency at which it was applied in the study might be insufficient to neutralize its activity.

### Galectin-3 and Galectin-3 Inhibitors

Galectin-3 is a β-galactoside-binding animal lectin in the nucleus and cytoplasm and on the cell surface that has been implicated in a variety of biological processes, including cell proliferation, survival and inflammation. Galectin-3 expression is upregulated in human fibrotic liver disease, and the level is associated with the induction and resolution of hepatic fibrosis in animal models (177). A mechanistic study showed that galectin-3 in HSC is required for TGF-β-mediated myofibroblast activation and matrix production during disease progression (177). Preclinical results showed that the galectin-3 inhibitor Belapectin (GR-MD-02) was effective in mouse models of NASH with advanced fibrosis or cirrhosis (178). Although Belapectin was safe and well-tolerated in a phase 1 trial (179), in this 16-week phase 2 clinical study, Belapectin treatment failed to alleviate liver fibrosis in patients with NASH with advanced fibrosis, as measured by multiparametric MRI corrected T1 mapping (NCT02421094). Similarly, another phase 2b trial of the safety and efficacy of Belapectin in patients with NASH, cirrhosis, and portal hypertension further showed that Belapectin was not associated with a significant reduction in the hepatic venous pressure gradient or fibrosis (NCT02462967) (180). However, treatment with Belapectin reduced the venous pressure gradient in a subset of patients without esophageal varices (180). To confirm this discovery, a phase 2b/3 trial evaluating the efficacy of Belapectin for the prevention of esophageal varices in NASH-associated cirrhosis was initiated and is expected to be completed in 2023 (NCT04365868).

## TGF-β and TGF-β Monoclonal Antibody

TGF-β is an important pleiotropic cytokine involved in many biological processes such as cell survival, proliferation, differentiation, angiogenesis, and wound healing (181). In advanced MAFLD, TGF-β is activated by HSCs, triggering a series of responses including tissue repair, extracellular matrix production, growth regulation, and apoptosis, ultimately leading to liver fibrosis (182). Studies in NASH model of wild-type and hepatocellular specific TGF-β receptor type II deficiency mice demonstrated that TGF-β signaling in hepatocytes promotes lipid accumulation by regulating lipid metabolism and enhancing cell death in hepatocytes that accumulate lipid, leading to the development of hepatic steatosis, inflammation, and fibrosis (183). Previous studies have shown a significant increase in TGF-β expression in the liver of patients with NASH fibrosis (184). Preclinical results showed that TGF-β inhibitor Galunisertib affected parenchymal cell fate by regulating the biochemical composition of deposited extracellular matrix and inhibited the progression of liver fibrosis, but did not significantly improve the pathological grading of fibrosis in Abcb4ko mice (185). At present, there have been clinical studies on TGF-β inhibitors restraining fibrosis in other organs (idiopathic pulmonary fibrosis and myelofibrosis), but there are no clinical studies related to liver fibrosis. It is believed that researchers will conduct many clinical studies on TGF-β inhibitors in the fibrosis process of MAFLD in the future.

## Conclusion and Perspectives

The burden of MAFLD/NAFLD is increasing rapidly with the ongoing metabolic disease epidemic (6, 7). MAFLD/NAFLD have shared and predominate causes from nutrition overload to persistent liver damage and eventually lead to the development of liver fibrosis and cirrhosis (10, 186). Discoveries have revealed that the pathogenesis of fibrosis in NAFLD involves multiple mechanisms and factors, such as lipid metabolism, inflammation, cell apoptosis, oxidative stress, extracellular matrix formation, and intestinal flora, as well as genetic and epigenetic regulation (17, 134). Reagents specifically targeting these pathways and receptor/ligand interactions have been developed, including agents acting on lipid synthesis, i.e., GLP-1 agonists, ACC inhibitors, FXR agonists, PPAR-α/δ agonists, and THR-β agonists, agents acting on cell stress and apoptosis, i.e., vitamin E and caspase inhibitors, agents acting on the innate immune system and inflammation, i.e., ASK1 inhibitors, TLR4 inhibitors, VAP-1 inhibitors, and CCR2/5 antagonists, and agents acting on other mechanisms, i.e., LOXL2 monoclonal antibodies and galectin-3 inhibitors (Supplementary Table 1). Although substantial advances have been made in the development of novel antifibrotic targets and therapeutic compounds, very few have reached clinical primary endpoints without significant side effects in large clinical studies. Therefore, it is important to recognize the boundaries and drawbacks of the traditional paths of drug discovery. First, since the majority of mechanistic investigations are based on rodent models, the application of models in large animals, such as non-human primates, with a closer resemblance to humans in the preclinical phase would likely allow for a higher chance of translating basic discoveries to clinical practice. Second, as the pathways involved in fibrosis are complex and targeting one mechanism may trigger alternative compensatory mechanisms, combination therapies targeting multiple profibrotic pathways could be promising in achieving successful antifibrotic interventions in patients with MAFLD/NAFLD. Third, the inconsistent results in previous trials have indicated that there may be large variations in the genetic predisposition and mechanisms involved in the pathogenesis of MAFLD/NAFLD fibrosis among individuals. A one-size-fits-all strategy would not be applicable for the treatment of MAFLD/NAFLD. Fourth, although liver biopsy is the gold standard for diagnosing NASH and assessing the stage of fibrosis in patients with NAFLD, this methodology misses the systemic evaluation of liver pathology and involves interobserver variations and biopsy bias. Liver biopsy cannot be performed for screening and follow-up in large populations due to its well-known limitations. There is an urgent need to improve the methodology in the evaluation of liver fibrosis.

In summary, the high prevalence of MAFLD/NAFLD paired with end-stage complications emphasizes the need for the discovery of effective and safe pharmaceutical treatments. In the current situation, one should keep in mind that appropriate lifestyle interventions with improvements in metabolic risk factors can potentially impede the development of MAFLD/NAFLD (13). In addition to accelerating the discovery of new pharmacotherapeutics, personalized medicine, combination therapies targeting multiple profibrotic pathways, and different methodologies for evaluating fibrosis would be beneficial for the development of new treatment strategies with good tolerability and efficacy.

## Author Contributions

WQ and TM collected the data and drafted the first edition of the paper. All authors listed have made a substantial, intellectual and direct contributions to this review, and authorized the publication of it.

## Funding

This work was supported by grants from the National Key R&D Program of China (2016YFF0101504, 2020YFC2004702), the National Science Foundation of China (81630011, 81970364, 81970070, 81770053, 81870171, and 81970011), the Hubei Science and Technology Support Project (2019BFC582, 2018BEC473), and Medical flight plan of Wuhan University (TFJH2018006).

## Conflict of Interest

The authors declare that the research was conducted in the absence of any commercial or financial relationships that could be construed as a potential conflict of interest.

## Publisher's Note

All claims expressed in this article are solely those of the authors and do not necessarily represent those of their affiliated organizations, or those of the publisher, the editors and the reviewers. Any product that may be evaluated in this article, or claim that may be made by its manufacturer, is not guaranteed or endorsed by the publisher.
