# Supplementary material for: Liver Fibrosis and MAFLD: From Molecular Aspects to Novel Pharmacological Strategies
Source: Front Med (Lausanne). 2021 Oct 22;8:761538. doi: 10.3389/fmed.2021.761538 (PMC8568774; doi:10.3389/fmed.2021.761538)
Supplement: Supplementary file 1 [file Data_Sheet_1.DOCX]

| **Therapeutic targets** | **Agents** | **Phase** | **Patients** | **Primary Endpoint(s)** | **Trial success/fail** | **Adverse Event** | **Duration** | **NCT number** |
| --- | --- | --- | --- | --- | --- | --- | --- | --- |
| **Targeting fibrosis driven from lipid synthesis** | | | | | | | | |
| GLP-1 agonist | Liraglutide | Phase 2 | 52 patients with NAS ≥ 3, obesity and Type 2 Diabetes Mellitu**s** (T2DM) | Liver inflammation and fibrosis by biopsy | Success | Mild to moderate gastrointestinal reactions | 48 weeks | [NCT01237119](https://clinicaltrials.gov/ct2/show/study/NCT01237119) |
|  | Exenatide | Phase 4 | 76 patients with NASH and T2DM | Change in liver fat content (%) measured by MRS | Success | Hypoglycemic events | 24 weeks | [NCT02303730](https://clinicaltrials.gov/ct2/show/record/NCT02303730?cond=NCT02303730&draw=2&rank=1) |
|  | Semaglutide | Phase 2 | 320 patients with biopsyconfirmed NASH, liver fibrosis of stage F1, F2, or F3. | Resolution of NASH with no worsening of fibrosis | Success | Nausea, constipation, and vomiting | 72 weeks | [NCT02970942](https://clinicaltrials.gov/ct2/show/NCT02970942?cond=NCT02970942&draw=1&rank=1) |
|  | Cotadutide | Phase 2 | 74 obese subjects with NAFLD/NASH | Evaluate the safety and pharmacodynamic effects | *Data not published* | *Data not published* | 19 weeks | [NCT04019561](https://clinicaltrials.gov/ct2/show/NCT04019561?cond=NCT04019561&draw=2&rank=1) |
|  | Tirzepatide | Phase 2 | 318 patients with T2DM | Change in Hemoglobin A1c (HbA1c) Bayesian Dose Response | Success | Diarrhoea, nausea, and vomiting | 26 weeks | [NCT03131687](https://clinicaltrials.gov/ct2/show/results/NCT03131687?cond=NCT03131687&draw=2&rank=1) |
| ACC inhibitor | Firsocostat (GS-0976/NDI-010976) | Phase 2 | 127 subjects with Nonalcoholic Steatohepatitis of at least 8% | Measures of hepatic steatosis, stiffness, serum markers of fibrosis, and plasma metabolomics were evaluated. | Success | Mild to moderate gastrointestinal reactions | 12 weeks | [NCT02856555](https://clinicaltrials.gov/ct2/show/NCT02856555?cond=NCT02856555&draw=2&rank=1) |
|  | Cilofexor /**Firsocostat** | Phase 2b | 392 patients with bridging fibrosis or compensated cirrhosis (F3-F4) | Proportion of ≥1-stage improvement in fibrosis without worsening of NASH | Fail | Pruritus, upper respiratory tract infection, and nausea | 48 weeks | [NCT03449446](https://clinicaltrials.gov/ct2/show/results/NCT03449446?cond=NCT03449446&draw=1&rank=1&view=results) |

**Table 1. Molecular targets and related drugs in MAFLD/NASH fibrosis.**

| **Therapeutic targets** | **Agents** | **Phase** | **Patients** | **Primary Endpoint(s)** | **Trial success/fail** | **Adverse Event** | **Duration** | **NCT number** |
| --- | --- | --- | --- | --- | --- | --- | --- | --- |
| ACC inhibitor | PF-05221304 | Phase 2 | 450 patients with NASH and liver fibrosis of stage F3 or F4. | Resolution of NASH without worsening of fibrosis or improvement in fibrosis by ≥1 or both | Recruiting | / | 48 weeks | [NCT04321031](https://clinicaltrials.gov/ct2/show/NCT04321031?cond=NCT04321031&draw=2&rank=1) |
| FASN inhibitor | TVB-2640 | Phase 1/2 | 13 subjects with characteristics of the metabolic syndrome | Change in hepatic lipogenesis | Success | Alopecia | 10 days | [NCT02948569](https://clinicaltrials.gov/ct2/show/NCT02948569) |
|  |  | Phase 2 | 141 subjects with non-alcoholic steatohepatitis | The change in hepatic fat fraction by proton-density fat fraction by magnetic resonance imaging | Recruiting | / | 12 weeks | [NCT03938246](https://clinicaltrials.gov/ct2/show/NCT03938246) |
|  |  | Phase 2b | 330 subjects with liver fibrosis stage F2-F3 | Histological reduction in NAS ≥2 points that results from reduction of necro-inflammation or improvement in liver fibrosis | Recruiting | / | 52 weeks | [NCT04906421](https://clinicaltrials.gov/ct2/show/NCT04906421?cond=NCT04906421&draw=2&rank=1) |
| SCD1 inhibitor | Aramchol | Phase 2 | 60 patients with steatosis due to NAFLD or NASH | The difference between initial and final liver triglyceride concentration | Success | Without significant adverse effects | 3 months | [NCT01094158](https://clinicaltrials.gov/ct2/show/NCT01094158?term=NCT01094158&draw=2&rank=1) |
|  |  | Phase 2 | 247 patients with NASH | Changes of mean liver fat as measured by MRS | Success | Nausea, constipation, vomiting | 52 weeks | [NCT02279524](https://clinicaltrials.gov/ct2/show/results/NCT02279524?cond=NCT02279524&draw=2&rank=1&view=results) |
|  |  | Phase 3 | 2000 NASH patients with stage 2-3 fibrosis and metabolic disorders | Proportion of subjects with improvement in fibrosis ≥1 and no worsening of steatohepatitis | Recruiting | / | 72 or 120 weeks | [NCT04104321](https://clinicaltrials.gov/ct2/show/NCT04104321?cond=NCT04104321&draw=2&rank=1) |

| **Therapeutic targets** | **Agents** | **Phase** | **Patients** | **Primary Endpoint(s)** | **Trial success/fail** | **Adverse Event** | **Duration** | **NCT number** |
| --- | --- | --- | --- | --- | --- | --- | --- | --- |
| FXR agonist | Obeticholic acid | Phase 2 | 283 adult patients with non-alcoholic steatohepatitis | Improvement in NAFLD score ≥ 2 without worsening of fibrosis | Success | Pruritus | 72 weeks | [NCT01265498](https://clinicaltrials.gov/ct2/show/NCT01265498?term=OCA%EF%BC%8CFLINT&draw=2&rank=1) |
|  |  | Phase 3 | 2480 adult patients with definite NASH, NAFLD activity score ≥4, and fibrosis stages F2–F3, or F1 with ≥1 accompanying comorbidity | Fibrosis improvement (≥1 stage) with no worsening of NASH, or NASH resolution with no worsening of fibrosis | Active, not recruiting | Pruritus | 7 years | [NCT02548351](https://clinicaltrials.gov/ct2/show/NCT02548351?term=NCT02548351&draw=2&rank=1) |
|  | Obeticholic acid | Phase 2 | 919 Subjects with compensated cirrhosis due to NASH | Percentage of subjects with improvement in fibrosis ≥1 with no worsening of NASH, using CRN scoring system | Active, not recruiting | / | 18 months | [NCT03439254](https://clinicaltrials.gov/ct2/results?cond=NCT03439254&term=&cntry=&state=&city=&dist=) |
|  | **Obeticholic Acid** /Atorvastatin | Phase 2 | 84 participants with NASH | The change of Low-density Lipoprotein (LDL) concentration | Success | Pruritus | 16 weeks | [NCT02633956](https://clinicaltrials.gov/ct2/show/NCT02633956?term=NCT02633956&draw=2&rank=1) |
|  | Cilofexor | Phase 2 | 140 subjects with noncirrhotic NASH | Percentage of participants experiencing TEAEs | Success | Moderate to severe pruritus | 24 weeks | [NCT02854605](https://clinicaltrials.gov/ct2/show/NCT02854605?cond=NCT02854605&draw=2&rank=1) |
|  | Selonsertib/ Firsocostat/**Cilofexor** | Phase 2 | 395 participants with ridging (F3) fibrosis or compensated cirrhosis (F4) due to NASH | Percentage of improvement in  fibrosis of ≥1 stage  without the worsening of NASH | Success | pruritus, upper respiratory tract infection, and nausea | 48 weeks | [NCT03449446](https://clinicaltrials.gov/ct2/show/NCT03449446?cond=NCT03449446&draw=2&rank=1) |
|  | **Tropifexor** /Cenicriviroc | Phase 2 | 193 adult patients with NASH | Number of participants with Adverse Events | *Data not published* | *Data not published* | 48 weeks | [NCT03517540](https://clinicaltrials.gov/ct2/show/NCT03517540?cond=NCT03517540&draw=2&rank=1) |
| **Therapeutic targets** | **Agents** | **Phase** | **Patients** | **Primary Endpoint(s)** | **Trial success/fail** | **Adverse Event** | **Duration** | **NCT number** |
| FXR agonist | **Tropifexor /**Licogliflozin | Phase 2 | 380adult patients with NASH and liver fibrosis | Whether the participant achieves in fibrosis of ≥1 stage without worsening of NASH | *Data not published* | *Data not published* | 48 weeks | [NCT04065841](https://clinicaltrials.gov/ct2/show/study/NCT04065841?term=Tropifexor&cond=liver+fibrosis&draw=2&rank=1) |
| FGF19 analogue | Aldafermin | Phase 2 | 254 patients with histologically confirmed NASH | Change in absolute liver fat content from baseline at week 24 | Success | Mild or moderate diarrhea, nausea, headache | 24 weeks | [NCT02443116](https://clinicaltrials.gov/ct2/show/NCT02443116?cond=NCT02443116.&draw=2&rank=1) |
|  |  | Phase 2b | 152 participants with histologically confirmed NASH | Proportion of participants achieving a histologic treatment effect as determined by the NASH CRN criteria | / | Active, not recruiting | 24 weeks | [NCT03912532](https://clinicaltrials.gov/ct2/show/study/NCT03912532?term=Aldafermin&cond=liver+fibrosis&draw=2&rank=1) |
|  |  | Phase 2 | 150 participants with compensated cirrhosis due to NASH | Percentage of improvement in  fibrosis of ≥1 stage  without the worsening of NASH | Recruiting | / | 48 weeks | [NCT04210245](https://clinicaltrials.gov/ct2/show/NCT04210245?term=NGM282&cond=NAFLD&draw=2&rank=3) |
| FGF21 analogue | Pegbelfermin | Phase 2 | 184 Adults participants with NASH | Mean Change in Percent Hepatic Fat Fraction (%) by MRI | Success | Diarrhoea, nausea | 16 weeks | [NCT02413372](https://clinicaltrials.gov/ct2/show/NCT02413372) |
|  |  | Phase 2 | 160 adults with NASH and stage 3 liver fibrosis | Proportion of participants with improvement in fibrosis ≥1 stage without worsening of NASH | Active, not recruiting | / | 24 weeks | [NCT03486899](https://clinicaltrials.gov/ct2/show/NCT03486899?cond=NCT03486899&draw=2&rank=1) |
|  |  | Phase 2 | 155 patients with NASH and compensated liver cirrhosis | Proportion of participants with improvement in fibrosis ≥1 stage without worsening of NASH | Active, not recruiting | / | 52 weeks | [NCT03486912](https://clinicaltrials.gov/ct2/show/NCT03486912?cond=NCT03486912&draw=2&rank=1) |
|  | Efruxifermin | Phase 2 | 110 subjects with NASH | Change from baseline in hepatic fat fraction assessed by MRI-PDFF | Active, not recruiting | / | 24 weeks | [NCT03976401](https://clinicaltrials.gov/ct2/show/NCT03976401) |

| **Therapeutic targets** | **Agents** | **Phase** | **Patients** | **Primary Endpoint(s)** | **Trial success/fail** | **Adverse Event** | **Duration** | **NCT number** |
| --- | --- | --- | --- | --- | --- | --- | --- | --- |
| PPARα agonist | Pirfenidone | Phase 2 | 281 patients with advanced liver fbrosis (ALF) | Fibrosis reversal based on Fibrotest | Success | Transient burning or nausea, and photosensitivity | 12 months | [NCT04099407](https://clinicaltrials.gov/ct2/show/NCT04099407?cond=NCT04099407&draw=2&rank=1) |
| PPARγ agonist | Pioglitazone | Phase 4 | 101 patients with prediabetes or T2DM and biopsy-proven NASH | Reduction of at  least 2 points in the nonalcoholic fatty liver disease activity score  without worsening of fibrosis. | Success | Few hypoglycemia | 18 months | [NCT00994682](https://clinicaltrials.gov/ct2/show/results/NCT00994682?cond=NCT00994682&draw=2&rank=1) |
|  | **Pioglitazone**/Vitamin E | Phase 4 | 105 hispanics with T2DM and NASH | Reduction of at  least 2 points in the nonalcoholic fatty liver disease activity score  without worsening of fibrosis. | Fail | Hypoglycemia. diarrhea/constipation | 18 months | [NCT01002547](https://clinicaltrials.gov/ct2/show/NCT01002547) |
|  | MSDC-0602K | Phase 2 | 392 patients with biopsy-confirmed NASH and fibrosis  (F1-F3) | Hepatic histological improvement of ≥2 points in NAS with a ≥1 point reduction in either ballooning or lobular inflammation and no increase in fibrosis stage | Fail | Non-cardiac chest pain | 52 weeks | [NCT02784444](https://clinicaltrials.gov/ct2/show/NCT02784444?cond=NCT02784444&draw=2&rank=1) |
| PPARα/δ agonist | Elafibranor (formerly GFT505) | Phase 2 | 276 patients with NASH without cirrhosis | Reversal of NASH without worsening of fibrosis. | Fail | Mild increase in serum creatinine | 52 weeks | [NCT01694849](https://clinicaltrials.gov/ct2/show/NCT01694849?cond=NCT01694849&draw=2&rank=1) |
| PPARα/γ agonist | Saroglitazar | Phase 2 | 106 patients with NAFLD and/or NASH | Percentage change in serum ALT levels | Success | No adverse events were directly linked to Saroglitazar | 32 weeks | [NCT03061721](https://clinicaltrials.gov/ct2/show/NCT03061721) |

| **Therapeutic targets** | **Agents** | **Phase** | **Patients** | **Primary Endpoint(s)** | **Trial success/fail** | **Adverse Event** | **Duration** | **NCT number** |
| --- | --- | --- | --- | --- | --- | --- | --- | --- |
| **Targeting fibrosis driven by cell stress and apoptosis** | | | | | | | | |
| Reactive oxygen species | Vitamin E | Phase 3 | 247 patients with NASH | Number of participants with improvement in NAFLD activity defined by change in standardized scoring of liver biopsies | Success | Weight gain | 96 weeks | [NCT00063622](https://clinicaltrials.gov/ct2/show/NCT00063622?cond=NCT00063622&draw=2&rank=1) |
|  |  | Phase 3 | 173 patients (aged 8-17 years) with biopsy-confirmed NAFLD | Sustained reduction in alanine  aminotransferase (ALT) | Fail | No adverse events were attributable to  treatment | 96 weeks | [NCT00063635](https://clinicaltrials.gov/ct2/results?cond=NCT00063635&term=&cntry=&state=&city=&dist=) |
|  | Pioglitazone/**Vitamin E** | Phase 4 | 105 hispanics with T2DM and NASH | Reduction of at  least 2 points in the nonalcoholic fatty liver disease activity score  without worsening of fibrosis. | Fail | Hypoglycemia. diarrhea/constipation | 18 months | [NCT01002547](https://clinicaltrials.gov/ct2/show/NCT01002547) |
|  | **Vitamin E** /Selenium | Phase 3 | 35533 healthy male volunteers | Number of participants with several cancers | Fail | Cardiac ischemia/infarction | 7-12 years | [NCT00006392](https://clinicaltrials.gov/ct2/show/study/NCT00006392) |
| Caspase inhibitors | Emricasan (IDN-6556) | Phase 2 | 38 subjects with NAFLD and raised transaminases | Change in ALT | Success | Bowel movements, chest pain, and headache | 28 days | [NCT02077374](https://clinicaltrials.gov/ct2/show/NCT02077374) |
|  |  | Phase 2 | 87 subjects with liver cirrhosis | Change from baseline at month 3 in cCK18/M30 | Success | Hepatic encephalopathy, ascites | 3 months | [NCT02230670](https://clinicaltrials.gov/ct2/show/NCT02230670) |
|  |  | Phase 2 | 318 subjects with NASH Fibrosis | Steatohepatitis resolution (based on liver biopsy) | Fail | Diarrhea, upper respiratory tract infection, nausea,  sinusitis | 72 weeks | [NCT02686762](https://clinicaltrials.gov/ct2/show/NCT02686762?cond=NCT02686762&draw=2&rank=1) |
|  |  | Phase 2 | 240 subjects with NASH cirrhosis and severe portal hypertension | Mean Change in Hepatic Venous Pressure Gradient | Fail | Edema peripheral, urinary tract infection, diarrhea, nausea | 48 weeks | [NCT02960204](https://clinicaltrials.gov/ct2/show/NCT02960204) |

| **Therapeutic targets** | **Agents** | **Phase** | **Patients** | **Primary Endpoint(s)** | **Trial success/fail** | **Adverse Event** | **Duration** | **NCT number** |
| --- | --- | --- | --- | --- | --- | --- | --- | --- |
| Caspase inhibitors | Emricasan (IDN-6556) | Phase 2 | 210 subjects with decompensated NASH cirrhosis | Any subject who died, had a new decompensation event, or an increase in MELD-Na score ≥4 points. | Fail | Adverse events of ascites | 120 weeks | [NCT03205345](https://clinicaltrials.gov/ct2/show/NCT03205345?cond=NCT03205345&draw=2&rank=1) |
| **Targeting fibrosis driven by the innate immune system and inflammation** | | | | | | | | |
| ASK1 inhibitor | Selonsertib | Phase 3 | 883 participants with compensated cirrhosis due to NASH | Percentage of participants who achieve a ≥ 1-stage improvement in fibrosis | Fail | hemorrhage | 240 weeks | [NCT03053063](https://clinicaltrials.gov/ct2/show/NCT03053063?cond=NCT03053063&draw=2&rank=1) |
|  | Selonsertib | Phase 3 | 808 subjects with NASH and bridging (F3) fibrosis | Percentage of participants who achieve a ≥ 1-stage improvement in fibrosis | Fail | Anaemia | 240 weeks | [NCT03053050](https://clinicaltrials.gov/ct2/show/NCT03053050?cond=NCT03053050&draw=2&rank=1) |
|  | **Selonsertib**/ Simtuzumab | Phase 2 | 72 participants with NASH and fibrosis stages F2-F3 | Number of participants who experienced TEAEs, SAEs, and any grade ≥ 1 laboratory abnormality | Success | Headache, nausea, sinusitis, nasopharyngitis,  upper abdominal pain, back pain | 24 weeks | [NCT02466516](https://clinicaltrials.gov/ct2/show/NCT02466516?cond=NCT02466516&draw=2&rank=1) |
|  | **Selonsertib**/ Firsocostat/Cilofexor | Phase 2 | 395 participants with ridging (F3) fibrosis or compensated cirrhosis (F4) due to NASH | Percentage of improvement in  fibrosis of ≥1 stage  without the worsening of NASH | Success | Pruritus, upper respiratory tract infection, and nausea | 48 weeks | [NCT03449446](https://clinicaltrials.gov/ct2/show/NCT03449446?cond=NCT03449446&draw=2&rank=1) |
| TLR4 inhibitors | JKB-121 | Phase 2 | 65 participants with NASH | Analysis of MRI-PDFF Change From Baseline to Week 24 | Success | Nausea, diarrhoea, and vomiting | 24 weeks | [NCT02442687](https://clinicaltrials.gov/ct2/show/results/NCT02442687?cond=JKB&draw=2&rank=2) |
|  | JKB-122 | Phase 2 | 300 participants with non-cirrhotic NASH and fibrosis | **T**he effect on liver histology | Not yet recruiting | / | 52 weeks | [NCT04255069](https://clinicaltrials.gov/ct2/show/NCT04255069?cond=JKB&draw=2&rank=5) |

| **Therapeutic targets** | **Agents** | **Phase** | **Patients** | **Primary Endpoint(s)** | **Trial success/fail** | **Adverse Event** | **Duration** | **NCT number** |
| --- | --- | --- | --- | --- | --- | --- | --- | --- |
| CCR antagonists | Cenicriviroc | Phase 3 | 1779 adult subjects with NASH | Proportion of subjects with improvement in fibrosis ≥1stage (NASH CRN system) and no worsening of steatohepatitis | Fail (This study was terminated early due to lack of efficacy) | / | 12 months | [NCT03028740](https://clinicaltrials.gov/ct2/show/NCT03028740?cond=NCT03028740&draw=2&rank=1) |
|  | Tropifexor /**Cenicriviroc** | Phase 2 | 193 adult patients with NASH | Number of participants with Adverse Events | *Data not published* | *Data not published* | 48 weeks | [NCT03517540](https://clinicaltrials.gov/ct2/show/NCT03517540?cond=NCT03517540&draw=2&rank=1) |
| **Targeting fibrosis driven by other mechanisms** | | | | | | | | |
| LOXL2 monoclonal antibody | Simtuzumab | Phase 2 | 222 participants with advanced liver fibrosis nut not cirrhosis secondary to NASH | Change from baseline to week 96 in hepatic collagen content, measured by morphometry of liver specimens | Fail (terminated early) | Hyperglycemia, ascites encephalopathy,  newly diagnosed varices, variceal hemorrhage | 96 weeks | [NCT01672866](https://clinicaltrials.gov/ct2/show/NCT01672866?cond=NCT01672866&draw=2&rank=1) |
|  | Simtuzumab | Phase 2 | 259 participants with compensated cirrhosis secondary to NASH | Change from baseline in hepatic venous pressure gradient (HVPG) | Fail (terminated early) | Headache, fatigue, diarrhoea, nausea | 96 weeks | [NCT01672879](https://clinicaltrials.gov/ct2/show/results/NCT01672879?cond=NCT01672879&draw=2&rank=1&view=results) |
| Galectin-3 inhibitors | Belapectin (GR-MD-02) | Phase 1 | 30 participants with NASH with advanced fibrosis | Safety and pharmacokinetics | Success | Headache and gout | 16 weeks | [NCT02421094](https://clinicaltrials.gov/ct2/show/study/NCT02421094?cond=GR-MD-02&draw=2&rank=6) |
|  |  | Phase 2 | 162 patients with nash cirrhosis | The Baseline-adjusted Mean Change in the Collagen Proportional Area | Fail | Nausea, diarrhoea, Abdominal pain upper | 52 weeks | [NCT02462967](https://clinicaltrials.gov/ct2/show/results/NCT02462967?cond=GR-MD-02&draw=2&rank=8) |
|  |  | Phase 2b/3 | 1010 patients with NASH cirrhosis and clinical signs of portal hypertension but without esophageal varices at baseline. | Proportion of patients in the belapectin treatment groups who develop new esophageal varices at 78 weeks | Recruiting | / | 78 weeks | [NCT04365868](https://clinicaltrials.gov/ct2/show/NCT04365868?cond=NCT04365868&draw=1&rank=1) |

**Abbreviations:** ALF, advanced liver fbrosis; ALT, alanine aminotransferase; CRN, Clinical Research Network; HVPG, hepatic venous pressure gradient; LDL, Low-density Lipoprotein ; LDL-C, Low-density Lipoprotein Cholesterol; MELD-Na, Model for End-stage Liver Disease; MRI-PDFF, Magnetic resonance imaging-derived proton density fat fraction; MRS, Magnetic Resonance Spectroscopy; NAS, NAFLD activity score; NASH, nonalcoholic steatohepatitis; SAEs, Serious Adverse Events; T2DM, Type 2 Diabetes Mellitu**s**; TEAEs, Treatment-Emergent Adverse Events.
